# Impact of barriers on Cyrno‐Sardinian endemisms: A comparative study of population genetics and phylogeography within taxa of *Centranthus* sect. *Nervosae* (Caprifoliaceae)

**DOI:** 10.1111/plb.13775

**Published:** 2025-03-10

**Authors:** O. De Castro, C. Piazza, E. Di Iorio, G. Bacchetta, B. Menale

**Affiliations:** ^1^ Department of Biology University of Naples Federico II Naples Italy; ^2^ Botanical Garden University of Naples Federico II Naples Italy; ^3^ Office of the Environment of Corsica (OEC) National Botanical Conservatory of Corsica (CBNC) Corte France; ^4^ Department of Life and Environmental Science, Center for Conservation of Biodiversity (CCB) University of Cagliari Cagliari Italy

**Keywords:** Blue island, endemism, Mediterranean, microsatellites, plastid sequences, polyploid, relict, SSR

## Abstract

- This study explores the impact of geographic barriers on the distribution and survival of Mediterranean endemic species, focusing on *Centranthus* sect. *Nervosae*, a tetraploid species complex found in Corsica and Sardinia. The aim is to analyse how these barriers influence genetic diversity, population structure, and phylogeographic pattern, thereby impacting conservation strategies and future resilience of the selected study species.
- Genotyping involved biparental markers (16 nuclear microsatellites for the population genetic survey) and Sanger sequencing of uniparental markers (six plastid sequences for the phylogeographic survey). Screening of microsatellites revealed a diploidisation process, and haplotype fixation in plastid sequence was observed across all populations.
- Results from both survey methods clearly indicate that isolation and barriers have significantly impacted the genetic structure of populations, subjecting them to genetic drift, bottlenecks and related evolutionary phenomena. Over time, these factors have resulted in the observed low haplotypic variability and nuclear microsatellite diversity.
- Reduced genetic variability, combined with factors such as inbreeding and genetic drift, highlight the vulnerability of these populations to extinction. Consequently, this multi‐approach survey has contributed to defining conservation strategies, stressing the need to preserve genetic diversity and mitigate the impacts of human activities and environmental changes on endemic plant communities in island‐like environments. The study emphasises the importance of integrating multiple marker types to deepen our understanding of conservation genetics and evolutionary history, thereby contributing to the assessment, and planning of potential safeguarding strategies for such endemic species.

## Graphical abstract

Ecological barriers such as mountains, promote the evolution of endemic species by preserving unique genetic traits and profoundly shaping their genetic structure through genetic drift, bottlenecks, and isolation.

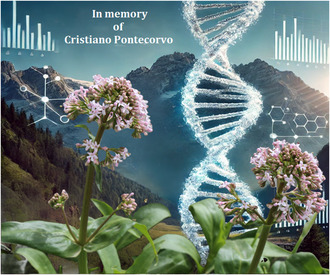

‘If we do not understand the history of the processes that have created this diversity and endemism, then we will never be able to grasp the conditions for its conservation’. [Thompson 2020]

## INTRODUCTION

### Biodiversity and endemism in the Mediterranean Basin

The Mediterranean Basin is one of the planet's largest biodiversity mega‐hotspots, hosting approximately 25,000 species of vascular plant with a high endemism rate, estimated at around 60%, half of which have a restricted distributions within so‐called “micro” and “nano” hotspots (Cañadas *et al*. 2014; Thompson 2020). These hotspots are primarily located on “blue islands”, small isolated geographic areas that harbour high biodiversity (Wilson 1992), often found on mountain peaks or isolated rocky outcrops. The islands of Sardinia and Corsica (the Cyrno‐Sardinian system) represent an intriguing environment in this regard, with numerous blue islands and an extraordinary diversity of endemic plants (Thompson 2005, 2020; Fois *et al*. 2022). These endemisms are the result of millions of years of adaptation to the specific environmental conditions of the islands (Thompson 2005, 2020). Their presence not only enriches the biological diversity of the Cyrno‐Sardinian system but also plays a crucial role in global biodiversity conservation. Species with endemic distributions such as these would not have been possible without the incidence of unique ecological barriers in these regions. Barriers, such as mountains and islands, can significantly contribute to the formation of plant endemisms; this arises because mountain ranges can isolate plant populations through the geographic barriers they create, including ridges, deep valleys and the varied climates encountered at different altitudes (Fenu *et al*. 2014; Noroozi *et al*. 2018; Thompson 2020). These factors can lead to the development of unique plant species adapted to the specific conditions of a mountainous area (e.g. *Petagnaea gussonei* (Spreng.) Rauschert and *Genista ephedroides* DC., De Castro *et al*. 2013, 2015; *Cunninghamia konishii* Hayata, Li *et al*. 2019; *Iris marsica* I.Ricci e Colas., De Castro *et al*. 2020a; *Veronica aragonensis* Stroh, Padilla‐García *et al*. 2021).

### *Centranthus* sect. *Nervosae* as a model for isolation and endemism

Species belonging to the former genus *Centranthus* DC. (now included in *Valeriana* L., Christenhusz *et al*. 2018), sect. *Nervosae* Rouy, represent an excellent model for studying isolation and endemism, yet remain poorly explored. The section currently consists of *C. amazonum* Fridl. & A.Raynal, *C. pontecorvi* Bacch. & Brullo present in Sardinia (Italy), and *C. trinervis* (Viv.) Bég from Corsica (France) (Bacchetta *et al*. 2024). This fascinating group of perennials, highly endemic, and rare rocky plants emerges as one of the most emblematic of these islands, offering the opportunity to examine the complex aspects of geographic isolation influencing their evolution and survival (Bacchetta *et al*. 2024). However, it is appropriate to specify that recently the genus *Centranthus*, considered synonymous with *Valeriana*, remains part of an unresolved evolutionary puzzle (De Castro *et al*. 2025). The phylogeny of *Valeriana* is far from being fully understood (Hidalgo *et al*. 2004; Bell & Donoghue 2005; Bell *et al*. 2012, 2015; Bell & Gonzalez 2018). Within this group, several monophyletic groups, including *Centranthus*, are recognised, each characterised by significant morphological autapomorphies (Hidalgo *et al*. 2004; Bell *et al*. 2015). Given the ongoing nature of studies and the potential for a comprehensive re‐evaluation of *Valeriana*, we currently adhere to the traditional concept of treating *Centranthus* as a genus (von Raab‐Straube 2017).

### Research objectives and conservation needs

In the current study, biparental nuclear microsatellite markers (nrSSRs) and uniparental plastid sequences (seq‐cpDNA) were employed to elucidate the evolutive history of the Cyrno‐Sardinian endemic taxa of *Centranthus*. This was achieved by adopting two principal approaches to interpret the gene flow via pollen (nrSSRs) and seeds (seq‐cpDNA), in order to delineate the genetic structure, diversity, and phylogeographic history of the *Centranthus* population under study. The objective was to investigate the mechanisms behind their present disjunct distribution and to evaluate their conservation status, thereby outlining priorities and strategies for their preservation. In light of this, the following goals were addressed: (i) elucidate the patterns of genetic connectivity among all documented populations of *Centranthus* taxa; (ii) estimate contemporary effective population sizes (Ne), providing essential insights for ongoing genomic analyses concerning the evolutionary history of these taxa; (iii) assess the extent to which barriers may have influenced the differentiation and formation of these endemic taxa; and finally, (iv) obtain information that will serve as the foundation for modelling their future evolutionary potential and long‐term viability within a changing environment.

Biodiversity is, indeed, profoundly threatened by anthropogenic disturbances compounded by climate change. Despite numerous studies on vascular plants (e.g. Freeland *et al*. 2012; De Castro *et al*. 2016; Gargiulo *et al*. 2018; Freeland 2020; Thompson 2020; Padilla‐García *et al*. 2021; Hanz *et al*. 2023), there remains an ongoing need for genetic studies to elucidate the mechanisms essential for effective conservation, whether *ex situ* or *in situ*.

## MATERIAL AND METHODS

### Study sites

Sardinia and Corsica are continental islands that form the Cyrno‐Sardinian microplate, a distinct geological unit that separated from the European plate during the Hercynian orogeny, ca. 300 Mya (Rosenbaum *et al*. 2002). This system is characterised by a complex geology, featuring Palaeozoic metamorphic and intrusive rocks, Mesozoic, and Cenozoic sedimentary rocks, and recent volcanics. Sardinia has an internal mountainous backbone composed of Palaeozoic rocks, surrounded by a hilly zone with more recent sediments. Corsica is more mountainous, with Monte Cinto exceeding 2,700 m and a granitic backbone. The formation of the Sardo‐Corso System occurred in several phases: the Hercynian orogeny led to the formation of mountains and rock metamorphism (Palaeozoic); deposition of marine and continental sediments (Mesozoic); collision with the African plate and the formation of the Apennines (Cenozoic); and the opening of the Tyrrhenian Sea and rotation of the Sardo‐Corso block (Cyrno‐Sardinian microplate) (Neogene) (Rosenbaum *et al*. 2002). Because of its geological peculiarities resulting from isolation as a micro‐continent, the Sardo‐Corso system represents a natural laboratory for the study of speciation phenomena with particular attention to endemisms (Mansion *et al*. 2008; Thompson 2020).

### Study plants

**Fig. 1:**
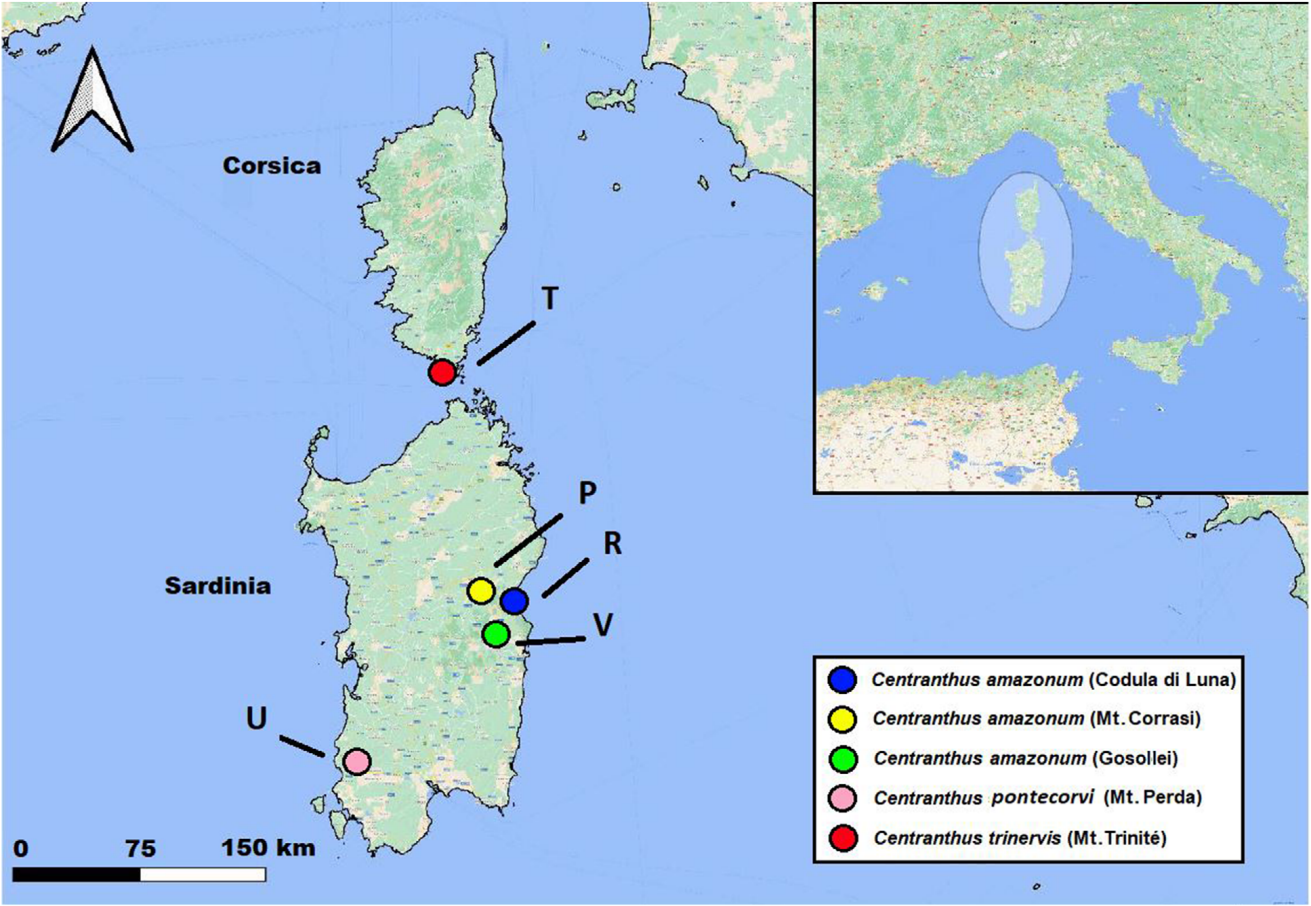
Map of sampling locations for *Centranthus* taxa sect. *Nervosae* analysed in this study. For further information, refer to Table 1.

**Fig. 2:**
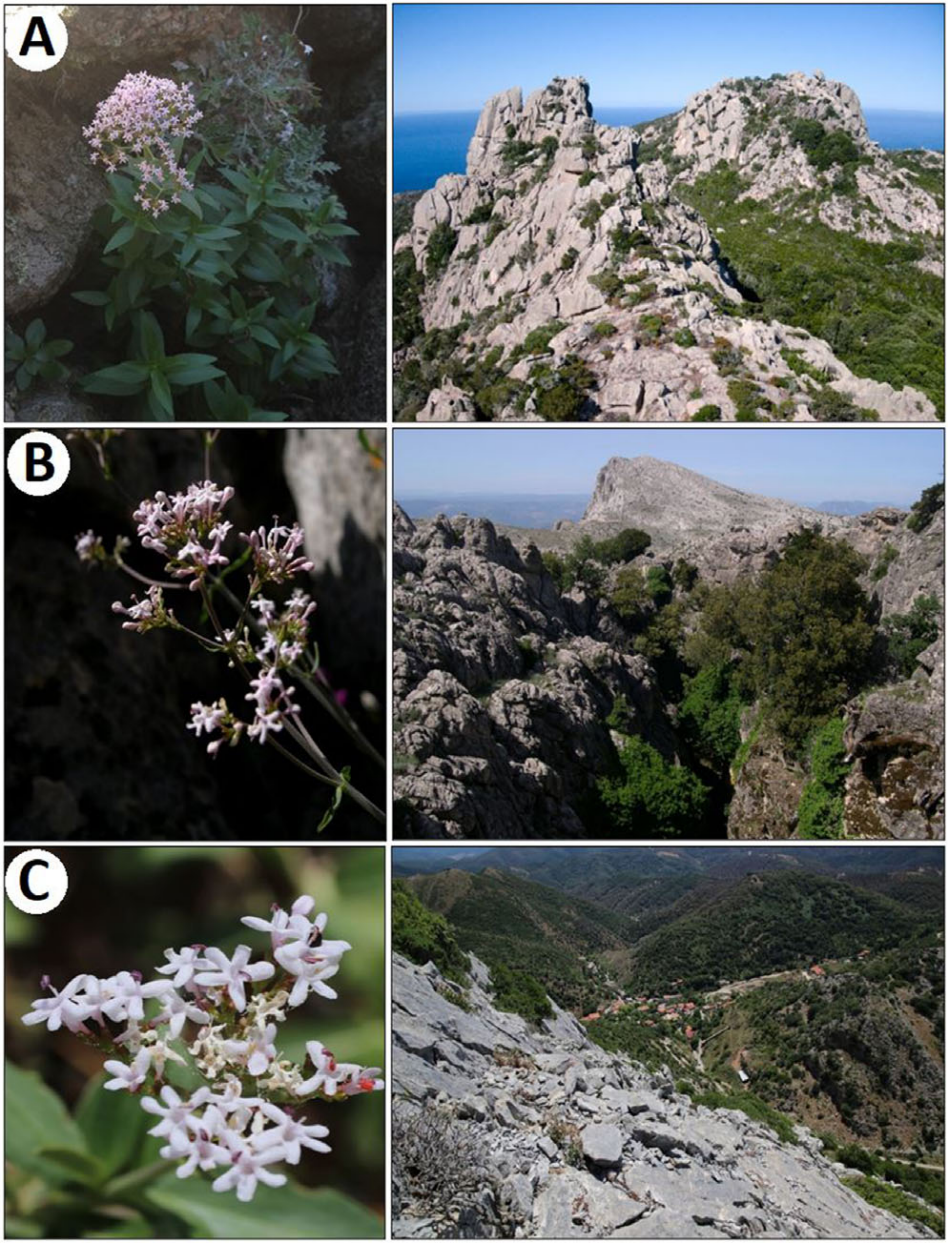
(A) Specimen of *Centranthus trinervis* at the granite massif of La Trinité in Bonifacio (Corsica; code T, Figure 1 and Table 1) (Photo by A. Delange, Y. Petit and C. Piazza). (B) Detail of the inflorescence of *C*. *amazonum* at the limestone relief of Mt. Corrasi (Sardinia; code P, Fig. 1 and Table 1) (Photo by G. Bacchetta). (C) Inflorescence of *C. pontecorvi* at the limestone complex of Mt. Perda (Sardinia; code U, Fig. 1 and Table 1) (Photo by G. Bacchetta).

*Centranthus trinervis* is found in Corsica at a unique location, with an estimated population of fewer than 150 specimens in the southern part of the island; these plants thrive exclusively on a granite formation of Palaeozoic origin, situated at elevations ranging from 140 to 200 m a.s.l. (Revaka *et al*. 2012) (Table 1, Figs. 1 and 2). In contrast, *C. amazonum* is endemic to central‐eastern Sardinia, featuring six populations comprising a very limited number of individuals (<102 mature specimens). The populations are located at elevations ranging from 165 to 1200 m a.s.l., in shaded areas and on Mesozoic calcareous‐dolomitic rocky substrates (Fridlender & Raynal‐Roques 1998; Bacchetta *et al*. 2008; Fenu *et al*. 2024) (Table 1, Figs. 1 and 2). Literature indicates that both *C. trinervis* and *C. amazonum* are tetraploid, possessing a karyotype of 2n = 4× = 32 (Contandriopoulos 1962; Corrias 1978; Contandriopoulos *et al*. 1987). No details are available regarding the type of polyploidy (i.e. autoploidy or allopolyploidy), except that this tetraploid pattern occurs in other taxa within the *Centranthus* genus and in closely related genera, such as *Fedia* Gaertn., *Valeriana*, and *Valerianella* Mill. (Hidalgo *et al*. 2010). The plants exhibit allogamy with the presence of protandry. Pollination is entomophilous, and wind plays a crucial role in seed dispersal through small achenes equipped with a pappus (Bacchetta *et al*. 2008). Vegetative reproduction is feasible via suckers and small stolons (Montmollin & Strahm 2005; Pasta *et al*. 2017). These species have been classified as CR (Critically Endangered) according to the IUCN Red List Criteria (IUCN 2023). Recently, a new taxon described from southern Sardinia belonging to the sect. *Nervosae*, *C. pontecorvi*, exhibits morphological and ecological uniqueness (Bacchetta *et al*. 2024). Currently, this species is identified in a single locality within southwestern Sardinia, hosting approximately 140 mature individuals. It is found at elevations between 565 and 715 m a.s.l., thriving on Cambrian metadolomites and metalimestones (Bacchetta *et al*. 2024) (Table 1, Figs. 1 and 2). No karyological data are available for this new taxon.

**Table 1:** Information on *Centranthus* sect. *Nervosae* populations sampled and related genetic indices, employing 16 nuclear microsatellite loci (nrSSRs) and haplotypes derived from six plastid sequences (seq‐cpDNA). The colours of the plastid haplotypes do not match those of the populations in the distribution map of the populations sampled (Fig. 1).

sampling						NrSSRs							plastid sequences			
code	taxon	*N*	country	locality	collectors (date) [voucher]^a^	L_M_	Na (SE)	Ne (SE)	A_R(10)_ (SE)	H_O_ (SE)	uHe (SE)	*F*_IS_	HT^b^ (*n*)			
													gap missing		gap 5th state	
T^1,2^	*Centranthus trinervis* (Viv.) Bég.	24	France (Corsica)	Mt. Trinité (Bonifacio)	C. Piazza, B. Berquez (21/06/2019) [CORS: Pva/CP/19062101, OEC]	8	1.5 (0.129)	1.332 (0.105)	1.467 (0.122)	0.133 (0.041)	0.188 (0.057)	0.297***	A		AA	
T_INT_^3^		11				12	1.25 (0.112)	1.241 (0.108)	1.25 (0.112)	0.108 (0.05)	0.129 (0.057)	0.167	A		AA	
T_AUT_^2^		13				8	1.5 (0.129)	1.297 (0.094)	1.497 (0.128)	0.154 (0.043)	0.182 (0.053)	0.16	A		AA	
P^2^	*C. amazonum* Fridl. & A.Raynal	10	Italy (Sardinia)	Mt. Corrasi (Oliena)	G. Bacchetta (28/07/2016) [G. Bacchetta 103/16, CAG]	6	1.875 (0.221)	1.526 (0.126)	1.875 (0.221)	0.269 (0.072)	0.285 (0.064)	0.06	**A**		**A**	
R^4^	*C. amazonum*	3	Italy (Sardinia)	Codula di Luna (Baunei)	G. Bacchetta (21/06/2018) [G. Bacchetta 03/18, CAG]	12	1.313 (0.12)	1.224 (0.089)	–	0.188 (0.074)	0.154 (0.06)	−0.286	**B**		**B**	
V^4^	*C. amazonum*	1	Italy (Sardinia)	Gosollei (Talana)	G. Fenu (05/08/2016) [G. Fenu 142/16, CAG]	–	–	–	–	–	–	–	**A**		**A**	
U^5^	*C*. *pontecorvi* Bacch. & Brullo	20	Italy (Sardinia)	Mt. Perda (Iglesias)	G. Bacchetta (24/06/2016) [G. Bacchetta, 77/16 CAG]	6	1.75 (0.171)	1.361 (0.097)	1.689 (0.152)	0.206 (0.053)	0.217 (0.052)	0.05	**C** (5)	**D** (15)	**C** (5)	**D** (15)

A_R_, allelic richness is the number of alleles independent of sample size as determined using the rarefaction method of El Mousadik and Petit (1996); *F*_IS_, inbreeding coefficient (Weir & Cockerham 1984) and the proportion of randomization that gave a larger F_IS_ value than that observed was also tested to show significant deviation of HWE (***P < 0.001); H_O_, observed heterozygosity; HT, typology of plastid haplotypes and the number of individuals exhibiting that haplotype, presented in parentheses; L_M_, monomorphic loci; N, number of individuals sampled per population; N_A_, different number of alleles; N_E_, effective number of alleles; T_AUT_, autochthonous individuals; T_INT_, introduced individuals, see point 3; uH_E_, unbiased expected heterozygosity.

a One voucher was selected to represent the population and deposited in the reference herbarium or public collection;

b polyN repeat exclude from the analyses; ^1^total population (T) with introduced (T_INT_) and autochthonous individuals (T_AUT_); ^2^one sample eliminated as clone after multilocus genotype screening using microsatellite markers; ^3^11 individuals introduced from ex‐situ project conservation (Project CARE‐MEDIFLORA‐Activity 2.1.2, 08/07/2019; Conservatoire Botanique National de Corse/Office de l'Environnement de la Corse); ^4^taxon with a sampling deficit.

### Plant sampling

Non‐destructive sampling was conducted from 2016 to 2019. Because of the challenging nature of the species' habitats (cliffs), the sampling efforts were directed towards capturing the distribution of populations as accurately as possible. One specimen per population has been deposited in both the Herbarium CAG and the Conservatoire Botanique National de Corse (CBNC).

*Centranthus trinervis* was sampled from a single locality in Corsica (code T; Table 1, Figs. 1 and 2), distinguishing between autochthonous individuals (code T_AUT_) and introduced individuals (code T_INT_) from an *ex‐situ* conservation project (Project CARE‐MEDIFLORA‐Activity 2.1.2, 08/07/2019; Conservatoire Botanique National de Corse/Office de l'Environnement de la Corse). In Sardinia, *C. amazonum* was sampled from the two known localities in the central‐eastern part of the island, along with a new neighbouring population consisting of a single individual (codes P, R, and U; Table 1, Figs. 1 and 2). Additionally, two populations were included in the analysis: a single individual of *C. amazonum* found near the other *C. amazonum* populations, and another population of the new taxon *C. pontecorvi* discovered in southwestern Sardinia (codes V and U; Table 1, Figs. 1 and 2).

### Genomic DNA extraction

In total, 61 individuals were sampled across five populations (Table 1, Fig. 1). Genomic DNA was isolated from dried leaves utilising the E.Z.N.A. Plant DNA DS Kit (Omega BIO‐TEK) following tissue pulverisation with an UltraCool GeneReady Homogeniser (Life Real). DNA quality was assessed using both the NanoReady Touch spectrophotometer (Life Real) and 1% agarose gel electrophoresis containing SafeView Nucleic Acid Stain (Applied Biological Materials) and visualised with the UVIdoc HD5 gel documentation system (UVITEC). DNA concentration was estimated by employing a Qubit dsDNA HS Assay Kit with the Qubit 3 Fluorometer (Invitrogen, Thermo Fisher Scientific).

## POPULATION GENETIC SURVEY

### Nuclear microsatellite (nrSSR) amplification and analysis

An SSR loci screening was conducted utilising the microsatellite library derived from *C. trinervis* (Di Iorio *et al*. 2024). PCR amplification was performed employing the M13(−21)‐tailed PCR method (Schuelke 2000), with the number of PCR cycles set at 30. PCRs were executed in a 10 μL mixture containing 6 ng genomic DNA, 0.04 μM M13(−21)‐tail+forward primer, 0.16 μM reverse primer, and a fluorescently labelled M13(−21)‐tail primer (5′‐TGT AAA ACG ACG GCC AGT‐3′). A high‐fidelity Phusion High‐Fidelity PCR Master Mix (ThermoFisher Scientific) was utilised as per the manufacturer's instructions to minimise stuttering (minor alterations in allele size during PCR). The primer sequences, annealing temperatures, amplicon sequences, and allele lengths are detailed in Appendix S1–Sheet 1.

Subsequently, 0.5 μL (ca. 5 ng) of the PCR product was combined with 12 μL Hi‐Di Formamide (Applied Biosystems, Thermo Fisher Scientific) and 0.4 μL GeneTrace 500 Plus LIZ (CarolinaBioSystems), then analysed using the 3130 Genetic Analyser (Applied Biosystems, Thermo Fisher Scientific).

Allele sizes were determined through the application of Peak Scanner v. 1 software (Applied Biosystems, Thermo Fisher Scientific) and GelQuest v. 3.5 software (SequentiX–Digital DNA Processing), with electrophoretic patterns manually scored as advised in DeWoody *et al*. (2006). Approximately three samples per population (excluding the V population) were reamplified in separate reactions to ensure accuracy in size scoring, and samples lacking amplicons were amplified three times to confirm the electrophoretic pattern. Four individuals were consistently utilised as additional reference standards on each plate to guarantee correct allele sizing. MICRO‐CHECKER v. 2.2.3 software (van Oosterhout *et al*. 2004) was employed to identify potential scoring errors, such as stuttering and allele dropout (reduced amplification efficiency in larger alleles compared to smaller alleles). Input files were produced using GenAlEx v. 6.503 (Peakall & Smouse 2012) and PGDSpider v. 2.1.1.5 software (Lischer & Excoffier 2012), with manual edits where necessary.

In two Sardinian localities, sampling was limited (refer to Discussion for details), and these were consequently excluded from some population genetic analyses. It is necessary to exercise caution when interpreting specific population genetic results from these areas. These populations comprise *C. amazonum* from Mt. Corrasi (code P, three samples; see Table 1) and Gosollei (code U, one specimen; see Table 1). Additionally, the *C. trinervis* population was analysed both as a single population and as two distinct subpopulations, consisting of the introduced and autochthonous individuals (codes T, T_TINT_, and T_AUT_, respectively; see Table 1).

### Interpretation of SSR banding patterns

Despite the tetraploid nature of our *Centranthus* accessions, observations derived from all allele scoring have revealed the presence of one or two alleles per locus, indicative of a diploidised pattern, rather than a multiallelic pattern (see Appendix S1–Sheet 2). This observation was also noted in populations analysed in a study concerning the library for *C. trinervis* and several species belonging to *Centranthus* (Di Iorio *et al*. 2024).

In this case, we exclude the possibility that this diploidised pattern is caused by a very high frequency of null alleles because, otherwise, we should have also observed a distinct pattern of homozygosity (i.e. the absence of alleles) (Clark & Schreier 2017; Padilla‐García *et al*. 2021). As reported in the Introduction, there is no information about the type of ploidy, and only speculations can be made on this diploidised–polyploid pattern. Specifically, a preliminary analysis was performed to evaluate deviations from Hardy–Weinberg equilibrium (HWE) expectations for the loci used, an analysis also conducted by Marques *et al*. (2016) for an allelic pattern comparable to ours. However, unlike Marques *et al*. (2016), who observed departures from HWE for individual loci, in our *Centranthus* dataset, this was not observed (Appendix S1–Sheet 6). Due to these preliminary data and the literature on analytical methods to be used on polyploids (Esselink *et al*. 2004; Catalán *et al*. 2006; Dufresne *et al*. 2014; Clark & Schreier 2017; Kolář 2021) and on study systems comparable to ours (Catalán *et al*. 2006; Segarra‐Moragues *et al*. 2007; Marques *et al*. 2016; Zhao *et al*. 2019; Shiposha *et al*. 2020; Padilla‐García *et al*. 2021), the obtained allelic data were treated as a diploid set through a codominant matrix.

Furthermore, considering the potentially hybridogenic nature of the genus as well as of *C. trinervis* (Richardson 1975; Revaka *et al*. 2012), the stable chromosomal complement in *Centranthus* and in closely related taxa (*e.g*., *Fedia*, *Valeriana* and *Valerianella*) (Hidalgo *et al*. 2010; Rice *et al*. 2015) is more realistically considered to be that of diploidised amphidiploids or, more simply, polyploids in which a diploidization event has occurred, although the mechanisms involved remain poorly defined (Wolfe 2001; Soltis *et al*. 2004; Tamayo‐Ordóñez *et al*. 2016; Li *et al*. 2021). From what has been reported, we have thus ruled out the hypothesis that the taxa have an autopolyploid origin (polysomic inheritance), as in this case, one would expect at least more than two alleles per locus in an individual, given the unlikelihood of a high number of null alleles being present. Moreover, it is improbable that an autotetraploid would not have undergone mutations or rearrangements in allele lengths since its formation.

### Genetic diversity

The evaluation of specimens possessing identical multilocus genotypes (MLGs) was conducted using MLGsim v. 2 software (Stenberg *et al*. 2003), employing both the HWE and the more conservative Fixation Index (F_IS_) models, with 1,000 simulations. This software was also utilised to assess the likelihood that the observed MLGs were either clones or the result of random mating, using the Psex value. To mitigate biases in genetic estimations, identified clones were excluded from subsequent analyses to ensure a unique MLG for each population.

Linkage disequilibrium (LD) for the loci was evaluated using a probability test implemented in GENEPOP v. 4.7.5 software (Raymond & Rousset 1995; Rousset 2008). A Holm‐Bonferroni (HB) correction was applied to account for multiple comparisons (Holm 1979), utilising the Holm‐Bonferroni sequential correction v. 1.3.1 (Gaetano 2018) with alpha set to 0.05.

To estimate null allele frequencies, two distinct analytical approaches were employed. The first utilised the Expectation Maximisation (EM) algorithm (Dempster *et al*. 1977) as implemented in the FreeNA software (Chapuis & Estoup 2007), with 10,000 bootstrap replications. The second, a superior method for detecting the frequency of null alleles, involved a Maximum Likelihood (ML) approach (Dabrowski *et al*. 2015) using ML‐NULLFREQ software (Kalinowski & Taper 2006). The influence of null alleles was considered significant if their frequency exceeded 0.2 (Dakin & Avise 2004; Chapuis & Estoup 2007).

The number of different alleles (Na), effective number of alleles (Ne), observed and unbiased expected heterozygosity (H_O_ and uH_E_) for each locus, and polymorphic information content (PIC) were calculated using GenAlEx and Cervus v. 3.0.7 software (Marshall *et al*. 1998; Kalinowski *et al*. 2007), respectively. Probability tests for deviation from HWE and inbreeding coefficient values (F_IS_; Weir & Cockerham 1984) for the loci were evaluated using GENEPOP with an HB correction.

For each population, GenAlEx was employed to perform the analyses mentioned above (i.e. Na, Ne, H_O_ and uH_E_). Allelic richness (AR) based on the smallest sample sizes (i.e. 10 samples, for the *C. amazonum* population from Mt. Corrasi, code P) and *F*_IS_ values (Weir & Cockerham 1984) were calculated using FSTAT v. 2.9.4 software (Goudet 2003). Deviations from HWE were also tested by examining the deficit of heterozygotes within populations, with significant departures indicated by F_IS_ values significantly greater than those observed.

### Population structure

The genetic structure of the populations was assessed using several approaches. Initially, an Analysis of Molecular Variance (AMOVA) was conducted with Arlequin v. 3.5.2.2 software (Excoffier *et al*. 2005) to determine the partitioning of total genetic variation at both intra‐ and inter‐population levels across the entire dataset. Subsequent analyses focused on (1) only *C. trinervis*, divided into two subsamples (T_INT_ and T_AUT_; Table 1); (2) all *Centranthus* populations from central‐east and southwestern Sardinia (*C. amazonum* and *C. pontecorvi*); and (3) only the *C. amazonum* populations. Statistical significance was assessed with 10,000 permutations.

Populations v. 1.2.32 software was employed to calculate Nei's genetic distance (Da; Nei & Chesser 1983) among individuals (Langella 2011), which was visualised through a Principal Components Analysis (PCoA) using GenAlEx via a covariance standardised distance approach.

To estimate the distribution of individuals among natural genetic groups (*K*), the dataset was analysed using a Bayesian model‐based clustering method with STRUCTURE v. 2.3.4 software (Pritchard *et al*. 2000; Evanno *et al*. 2005). Three analyses were carried out on different datasets as in the previous AMOVA analyses, namely: (1) all datasets; (2) only *C. amazonum* populations; and (3) *C. trinervis* subdivided into two subsamples of introduced and autochthonous samples (T_INT_ and T_AUT_). The informativeness of the sampling location data (*r*; Hubisz *et al*. 2009), allele frequencies distribution (*λ*; Evanno *et al*. 2005), and the Dirichlet parameter (*α*; Hubisz *et al*. 2009) were evaluated *a priori*. A preliminary estimate of the possible genetic clusters (*K*) was determined based on the PCoA and literature recommendations (Evanno *et al*. 2005; Patterson *et al*. 2006). Following various simulation sets, Bayesian analysis was conducted using the admixture model without locprior, assuming correlated allele frequencies, and with a variable Dirichlet parameter for all populations. Ten independent replicates per number of *K*s ranging from 1 to 8 were run for the complete dataset. Each run involved a burn‐in period of 100,000 iterations followed by a post‐burn‐in simulation of 200,000 iterations. Similar settings were applied for the other two analyses, except for a fixed alpha and *K* set to 6 and 5 for the *C. amazonum* and *C. trinervis* populations, respectively.

STRUCTURE HARVESTER web v. 0.6.94 software (Earl & von Holdt 2012) was utilised to assess the consistency of the STRUCTURE replicates and to identify the most likely number of *K*s for dividing individuals, using the empirical statistic Δ*K* (Evanno *et al*. 2005). The output files were then analysed with CLUMPP v. 1.1.2 software (Jakobsson & Rosenberg 2007) to calculate the average of the different runs with the same *K* (pairwise similarity H′) using the Greedy algorithm with 1,000 repeats. STRUCTURE PLOT v. 2 software was used to visualise the genotypic structure through histograms and the cluster membership coefficients (*Q*) (Ramasamy *et al*. 2014). Individuals were assigned to a cluster if their *Q* coefficient was higher than 70% (threshold ≥0.7).

Isolation‐by‐distance (IBD) (Wright 1943) was tested by performing a Mantel test (Mantel 1967) on the pairwise population *F*_ST_/(1−*F*_ST_) matrix (Slatkin 1995) against the natural logarithm of their geographic distances (Rousset 1997). The pairwise genetic matrix was obtained using Arlequin (nPr = 10,000), and the geographic distance matrix and the Mantel test (nPr = 999) were computed using GenAlEx. Localities with sampling deficits, specifically *C. amazonum* from Mt. Perda and the Gosollei locality (codes P and V; Table 1) were excluded.

### Demographic history

It is important to highlight that the results obtained here may be subject to potential distortions due to concrete limitations in the dataset used (i.e. the number of polymorphic loci and the small sample size). However, the extent of these potential distortions is mitigated by the advantages of obtaining even approximate estimates for understanding the demographic history of *Centranthus* populations, which is also beneficial for conservation purposes. Due to the limitations of sampling and polymorphic loci in the populations under examination, analyses were exclusively performed on three populations that demonstrated greater statistical robustness, namely, those with a minimum of 10 individuals and eight polymorphic loci.

The effective population size (Ne) was estimated using the single‐sample estimator implemented in NeEstimator v. 2.1 software (Do *et al*. 2014), with particular attention given to the small dataset. The linkage disequilibrium (LD) method is most effective for microsatellite datasets (loci >10, samples ≈50, Ne <200) but can still provide useful information with a sampling of only 25–30 individuals (Waples & Do 2010). Moreover, this method assumes isolated populations, which is a realistic assumption for our *Centranthus* populations. To avoid bias in estimating Ne for rare alleles, the threshold values below which rare alleles are not considered (PCrit) was set to 0.02. A 95% confidence interval was obtained using the jackknife procedure.

To infer potential genetic bottlenecks across different time scales, two methodological approaches were performed. To detect reductions in population size within the last 2–4Ne generations (Cornuet & Luikart 1996), BOTTLENECK v. 1.2.02 software (Piry *et al*. 1999) was used with 10,000 replicates. The one‐tailed Wilcoxon test was employed to detect an excess of expected heterozygosity (H_E_) compared to that expected under mutation‐drift equilibrium (H_EQ_). The Two‐Phase Model (TPM; Di Rienzo *et al*. 1994) was applied with a variance for TPM of 12% and a proportion of Stepwise Mutation Model (SMM) in TPM of 95%, as recommended for SSR data (Piry *et al*. 1999). Comparisons were also made with different values of SMM in TPM and its variance (90 and 10; 70 and 30, respectively). Analyses considering only SMM were also conducted.

Finally, to detect an older reduction in effective population size (>100 generations), calculations were performed using the modified Garza‐Williamson index (modG‐W) or *M* ratio test (Garza & Williamson 2001) with Arlequin. This index is sensitive to past reductions in population size, as rare alleles tend to be lost through genetic drift when a population experiences a bottleneck. The ratio is very small in populations that have undergone a size reduction (*M* < 0.68) and close to one in stationary populations (*M* > 0.8) (Garza & Williamson 2001).

## PHYLOGEOGRAPHIC SURVEY

### Plastid sequences and data analyses

Six variable plastid markers were employed on 59 samples after eliminating the two clonal specimens according to the SSRs results. The markers included five intergenic spacers (IGS): *trn*W^(CCA)^‐*trn*P^(UGG)^‐*psa*J, *trn*S^(GCU)^‐*trn*G^(UCC)^, *acc*D‐*psa*I, *rp*S15‐*ndh*F, *trn*H^(GUG)^‐*psb*A; and the 3′ portion of the *mat*K gene. This selection was made after a preliminary screening of 66 molecular markers from the library of Dong *et al*. (2012), Prince (2015), and other references (Appendix S1–Sheet 3).

The six molecular markers used, and their amplification information, are reported in Appendix S1–Sheet 4. PCRs were performed using approximately 6 ng genomic DNA, 0.25 μM of each primer, and Phire Plant Direct PCR Master Mix (Thermo Fisher Scientific). The cycling parameters for the PCRs were conducted according to the manufacturer's instructions for the PCR Master Mix. Amplicons were purified using PEG‐8000 precipitation (PEG 15%, 2.5 M NaCl) and sequenced using 0.5 μL 6.4 μM primer (3.2 pmol) in a 5 μL volume with the Bright Dye Terminator Cycle Sequencing Kit (iCloning). The amount of template in a cycle sequencing reaction was as per the manufacturer's instructions. The reactions were purified using the BigDye XTerminator Purification Kit (Applied Biosystems, Thermo Fisher Scientific), and 13 μL of template were run on an automated sequencer (3130 Genetic Analyser; Life Technologies, Thermo Fisher Scientific). The raw data were analysed using AB DNA Sequencing Analysis v. 5.2 software (Applied Biosystems, Thermo Fisher Scientific), edited, and assembled in Chromas Pro v. 2.1.8 software (Technelysium Pty Ltd). Alignments for each plastid marker were made using ClustalW implemented in BioEdit v. 7.2.5 software (Hall 1999). The resulting sequences have been submitted to GenBank for each haplotype per marker (Appendix S1–Sheet 10). Alignment for each marker is reported in Supplementary Files S2– S7. A geographic map was prepared to evaluate the distribution and frequency of haplotypes after concatenating all alignment matrices.

### Genealogical relationships

A statistical network was employed using the parsimony method (MP; Templeton *et al*. 1992) implemented in TCS v. 1.23 software (Clement *et al*. 2000) on the six concatenated alignment matrices. TCS was run with a parsimony connection limit of 95%. This method estimates an unrooted haplotype network and a 95% plausible set of all haplotype lineages within that network. Each insertion/deletion (indel) was coded as a single mutation event using the simple gap coding method described by Simmons *et al*. (2001), as implemented in FastGap v. 1.2 (Borchsenius 2009). Mononucleotide repeats (polyN) were excluded from the analysis due to their potential to produce artefacts because of possible homoplasy (Jarne & Lagoda 1996; Goldstein & Pollock 1997; Provan *et al*. 2001). An additional computation was also performed using two outgroup species to identify a probable ancestral haplotype. Following the previous phylogeny, *C. calcitrapae* (L.) Dufr. [Aritzo (NU), Sardinia, Italy; voucher, Bacchetta s.n. CAG] and *C. macrosiphon* Boiss. (Tetuan, Morocco; voucher APP‐45979) were used as outgroups. Outgroup GenBank references are reported in Appendix S1–Sheet 11. Where necessary, network ambiguities (loops) were resolved by following the guidelines proposed by Crandall & Templeton (1993) (see also Pfenninger & Posada 2002).

## RESULTS

### Population genetic survey

#### Nuclear microsatellites

In the testing of 100 loci from the *C. trinervis* SSR library (Di Iorio *et al*. 2024), 37 loci were selected and genotyped across five populations, encompassing a total of 61 specimens. Following the genotyping analyses, 21 loci were excluded from subsequent analyses because 12 were monomorphic across all populations and nine exhibited variable amplification efficiency. Among the 16 retained loci, no evidence of null alleles (no amplicon), stuttering, or allele dropout was observed (Appendix S1–Sheet 2). As previously reported, only a diploidised pattern was observed among the tested loci (Appendix S1–Sheet 2). As mentioned earlier, the *C. amazonum* specimens from the Codula di Luna and Gosollei localities (codes R and U, respectively, in Table 1) are treated with considerable caution compared to the others, and for some analyses, they are not considered due to being non‐representative because of a sampling deficit (one and three samples, respectively).

Two samples with identical Multilocus Genotypes (MLGs) were identified for *C. trinervis* (code T) and *C. amazonum* from Mt. Corrasi (code P) (Appendix S1–Sheet 2). One clone for each MLG was removed from the final dataset (Table 1). Based on the Psex values calculated with both models, the identical individuals were considered as clones and not a result of a sexual reproduction event for the two populations [Psex value for T/P populations = 0/3.7e‐14 (HW) and 3.33e‐11/1.128e‐9 (F_IS_), respectively; *P* < 0.0001].

The analyses of null allele frequencies (NAF) using the Expectation Maximisation (EM) algorithm indicated that the impact of total null alleles on the genetic analyses was negligible (Appendix S1–Sheet 5). The range of values was 0.0007 (SE ± 0.0001) in the *C. amazonum* population (code R) and 0.0372 (SE ± 0.016) in the only *C. trinervis* population (code T); per locus versus all populations, the maximum value of null allele frequency was approximately 0.04 for two loci (13 and 15); considering locus versus population, only two combinations had a value of 0.2 (locus 7 and 15 versus the *P* population). NAF results obtained using the Maximum Likelihood (ML) approach were congruent with those obtained by the EM algorithm (Appendix S1–Sheet 5).

#### Genetic diversity

The genetic parameters for each locus are shown in Appendix S1–Sheet 1, where the number of alleles detected per locus ranged from 2 to 7; the PIC index has a range of 0.107 (locus 7) to 0.717 (locus 10) with an average of 0.411 (SE ± 0.05). Evidence of non‐random association of alleles (LD) at different loci was found for a combination of loci in the *C. amazonum* (code P) (locus 10 versus 12); presence of LD was also observed in the *C. trinervis* population (code T) for one locus combination (locus 13 versus 16) if analysed totally, i.e. considering both introduced and autochthonous specimens. Deviations from random mating (HWE) were observed in two loci in *C. trinervis* considering it as a unique population (code T), i.e. with introduced and autochthonous individuals (Appendix S1–Sheet 6). A mixture of positive and negative F_IS_ for different loci was evident for the populations (Appendix S1–Sheet 6).

Values of the genetic diversity indices for each population are shown in Table 1. Globally, a small deficiency in heterozygotes was present in the populations analysed (uH_E_ >H_O_), except for R (heterozygote excess) and U (not evaluable) Sardinian accessions (Codula di Luna and Mt. Perda, respectively) which presented a sampling deficit. *Centranthus trinervis* exhibits less genetic diversity in terms of observed heterozygosity, also caused in part by the contribution of the individuals introduced (T_INT_). *Centranthus* Sardinian populations presented variability in terms of heterozygosity, with a range of 0.188 to 0.269 (R versus P populations). Inbreeding coefficient was relatively low except for the *C. trinervis* population (F_IS_ = 0.297; code T, Table 1) where a significant deviation from the coefficient of inbreeding was clearly observed. The other populations have shown no significant value of deviation, although the P and U populations were not statistically informative (Table 1). Allelic richness computed on the minimum sample size of ten diploid individuals was highest at the Mt. Corrasi location (*C. amazonum*, code P) and lower in the samples introduced from *C. trinervis* (T_INT_) (Table 1).

#### Population structure

Globally, the AMOVA revealed moderate genetic differentiation among individuals (36.06%), with the majority of variation being partitioned within populations (63.94%) (*F*_ST_ = 0.64, *P* < 0.0001). Conversely, when considering only the *C. trinervis* population divided into two sampling groups (T_INT_ versus T_AUT_), the source of variation was predominantly among individuals (72.62%), and the level of differentiation between the two sub‐populations was notably high (27.38%) (*F*_ST_ = 0.27, *P* < 0.0001). Additionally, a high level of differentiation among populations was observed, both with only *C. amazonum* taxa (55.73%) (*F*_ST_ = 0.56, *P* < 0.001) and including *C. pontecorvi* from southwestern Sardinia (60.73%) (*F*_ST_ = 0.61, *P* < 0.0001). Excluding the two locations due to a sampling deficit (*C. amazonum* from Codula di Luna and Gosollei, codes R and V), pairwise estimates of F_ST_ ranged from 0.587 to 0.646 (*C. amazonum*, code *P* versus *C. pontecorvi*, code U; *C. trinervis*, code T versus *C. pontecorvi*, code U), indicating very high levels of genetic differentiation across the populations, as previously demonstrated (Appendix S1–Sheet 7).

**Fig. 3:**
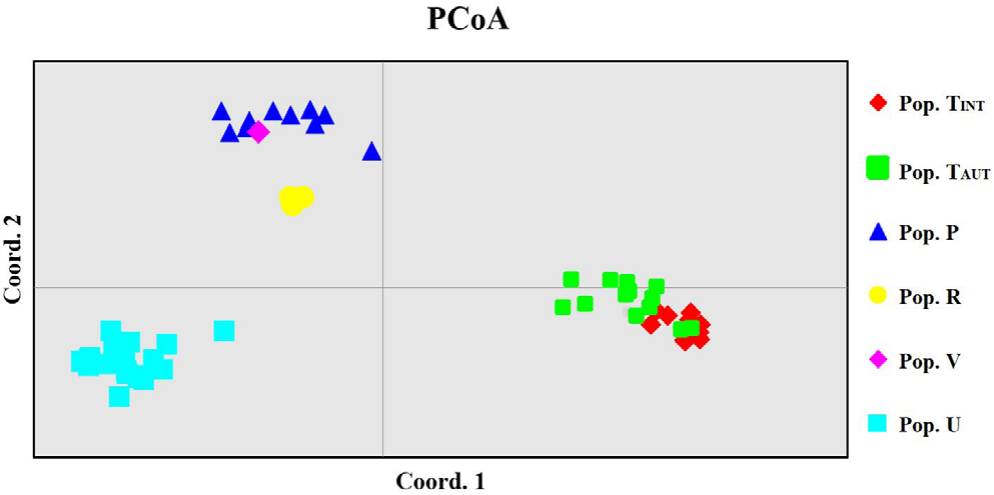
Principal Coordinates Analysis (PCoA) based on Nei's genetic distance (Da; Nei & Chesser 1983) on the 58 individuals of *Centranthus* analysed in this study. Both axes account for approximately 62.12% of the total genetic variation. For the code and geographic distribution of populations, refer to Table 1 and Fig. 1.

The PCoA using Da distance demonstrated a clear separation of the populations according to individuals, except for the single specimen of *C. amazonum* from Mt. Perda (code V); the first two axes explained 62.12% of the total genetic variation (1/2/3 axes = 38.98/23.14/9.1) (Fig. 3). Regarding *C. trinervis*, two sub‐groups were identified, corresponding to introduced versus autochthonous individuals (Fig. 3). The matrix of Nei's genetic distance (Da) among individuals is presented in Appendix S1–Sheet 8.

**Fig. 4:**
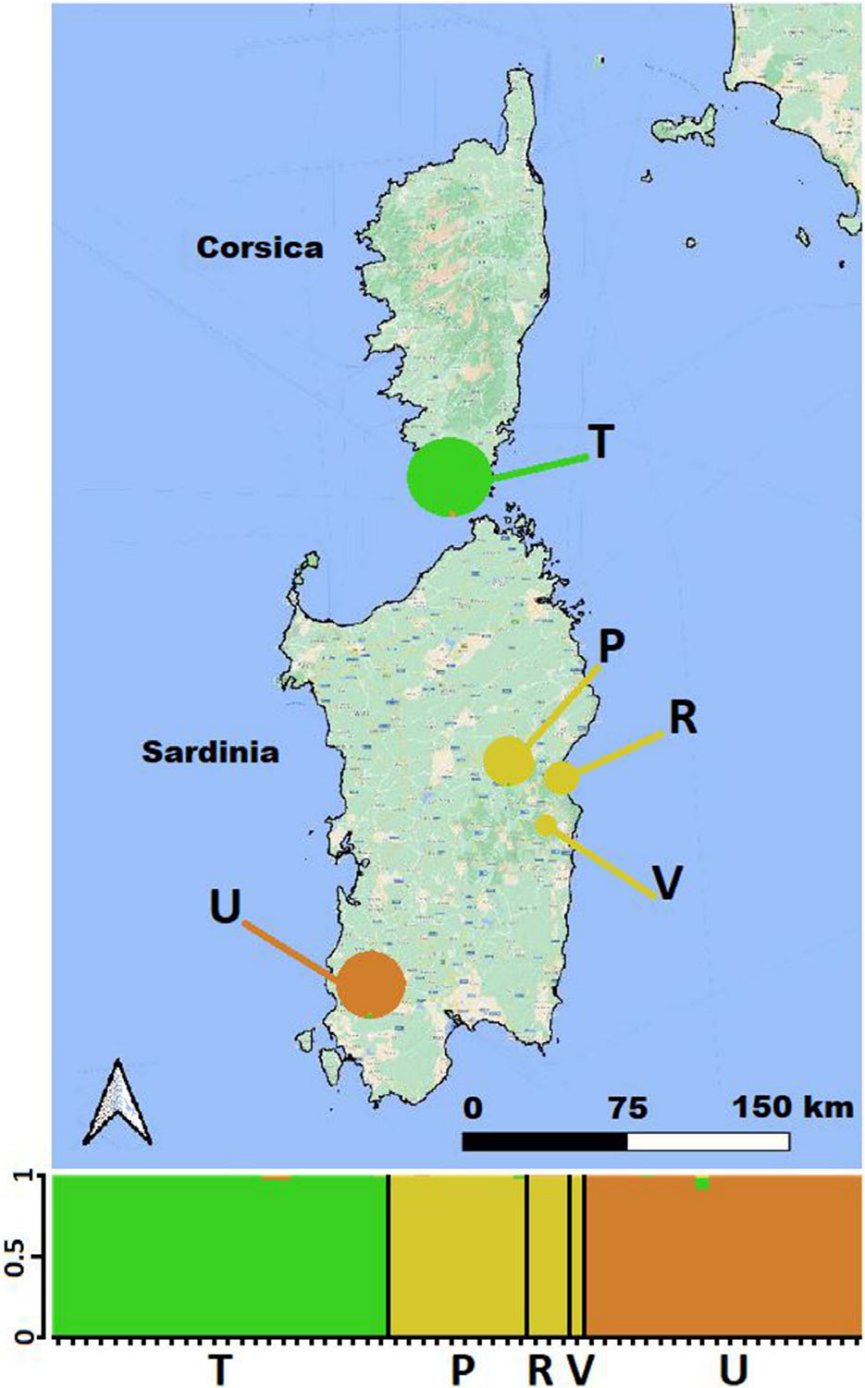
Genetic pools obtained through STRUCTURE analysis (*K* = 3) on the *Centranthus* populations under study. Each individual is represented by a vertical line on the *x*‐axis, whereas the length of each segment is proportional to the estimated membership of each genetic pool. The cluster membership coefficient (*Q*) is plotted on the *y‐*axis. For the population legends, refer to Table 1.

**Fig. 5:**
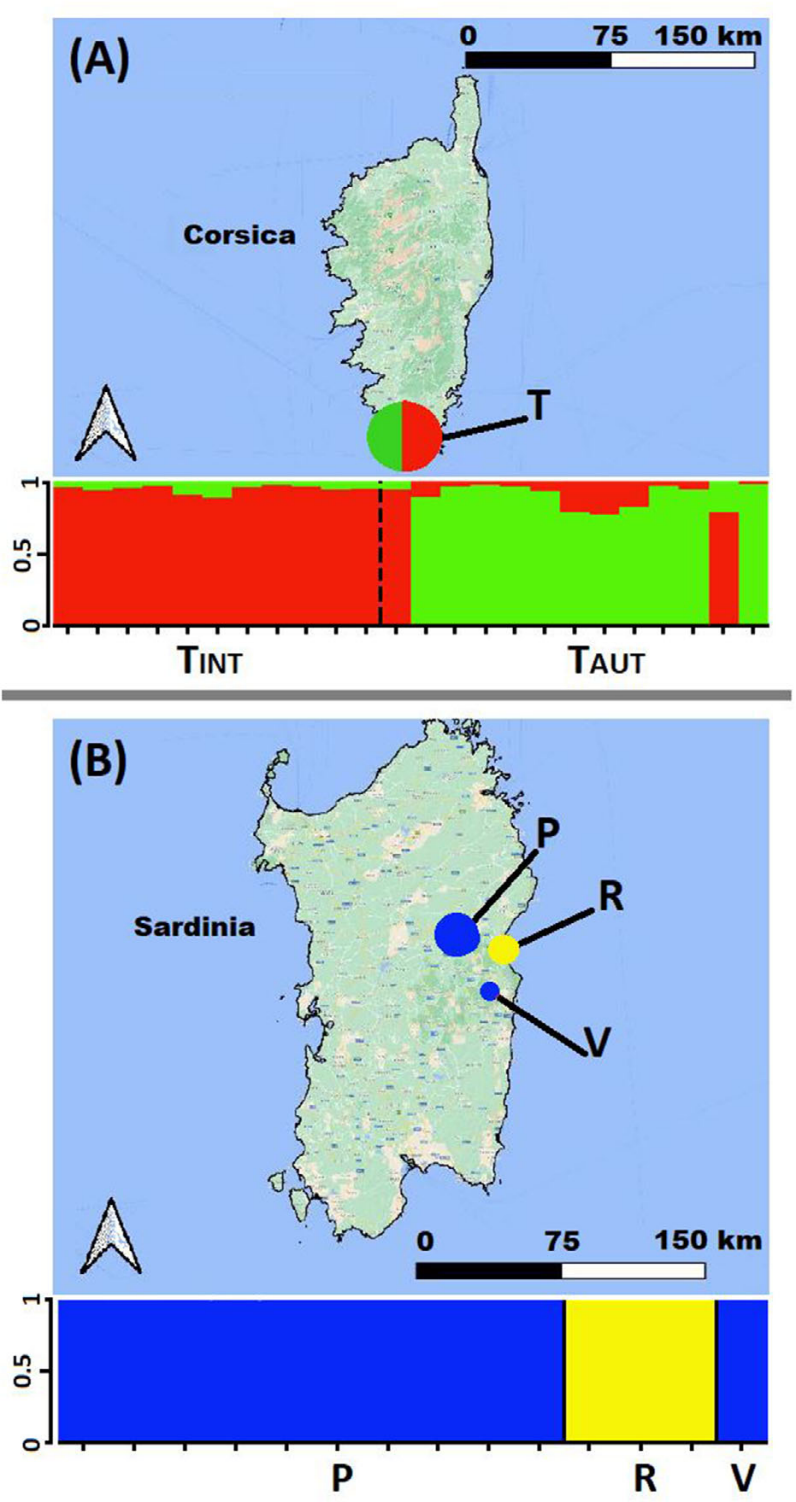
Genetic pools obtained through STRUCTURE analysis (*K* = 2) on two separate datasets: (A) the *C. trinervis* population, subdivided into sub‐samples of autochthonous (TAUT) and reintroduced (TINT) individuals; and (B) the populations of *C. amazonum*. Each individual is represented by a vertical line on the *x*‐axis, with each segment's length being proportional to the estimated membership within each genetic pool. The cluster membership coefficient (*Q*) is plotted on the *y*‐axis. For population legends, refer to Table 1.

Analysis of the entire dataset using a Bayesian clustering method implemented in STRUCTURE identified three distinct genetic clusters (*K* = 3), with no admixture observed among the genetic pools: one for *C. trinervis*, the second for *C. amazonum* populations, and the last for *C. pontecorvi* from southwestern Sardinia (Fig. 4; Appendix S1–Sheet 9). Taking into consideration analyses conducted across various hierarchical levels (refer to Materials and Methods), within the *C. trinervis* population there were two genetic clusters identified (*K* = 2); these correspond to the two subsampling groups comprising introduced individuals (T_INT_) along with two autochthonous individuals (T_AUT_) (Fig. 5A, Appendix S1–Sheet 9). Indeed, the dataset for *C. amazonum* populations also demonstrated two well‐defined genetic clusters (*K* = 2), delineating Mt. Corrasi and Gosollei from Codula di Luna (codes P + U versus R; Table 1, Fig. 5B, Appendix S1–Sheet 9).

Despite a high positive correlation between geographic and genetic distance (*r* = 0.75), the Isolation by Distance (IBD) test was not significant (*P* = 0.157).

#### Demographic history

Estimates of the effective population size (Ne) revealed markedly low values for *C. trinervis* (code T) and *C. amazonum* populations (code P), yielding 2.3 (95% CI = 1.5–4.3) and 1.6 (95% CI = 0.7–7.2), respectively (Table 2). For the *C. pontecorvi* population (code U), Ne was determined to be 90, albeit accompanied by a non‐informative confidence interval (6.7–∞) (Table 2).

**Table 2:** Results of demographic analyses in the three populations of *Centranthus* sect. *Nervosae*, which exhibited greater statistical robustness (i.e. ≥10 specimens and ≥8 polymorphic loci), are presented.

code	taxon	locality	Ne (95% CI)	Wilcoxon's sign‐rank test^a^		modG‐W (±SE)
				TPM	SMM	
T	*Centranthus trinervis*	Mt. Trinité (Bonifacio)—Corsica (FR)	2.3 (1.4–4.3)	0.014	0.02	0.137 (0.102)
P	*C. amazonum*	Mt. Corrasi (Oliena)—Sardinia (IT)	1.6 (0.7–7.2)	0.065	0.065	0.171 (0.123)
V	*C. pontecorvi*	Mt. Perda (Iglesias)—Sardinia (IT)	90 (6.7–∞)	0.449	0.482	0.153 (0.099)

CI, confidence interval estimated by jackknifing; modG‐W, modified Garza‐Williamson index; Ne, effective population size; SMM, stepwise mutation model; TPM, two‐phase mutation model.

a *P*‐values are shown for Wilcoxon's sign‐rank test under both the SMM and the TPM with SMM 95% in TPM and variance of 12%.

Wilcoxon's rank test indicated recent demographic bottlenecks under both the Stepwise Mutation Model (SMM) and the Two‐Phase Model (TPM) within the *C. trinervis* population (code T) (Table 2), with no such evidence in the two Sardinian populations, namely, *C. pontecorvi* (code U) and *C. amazonum* from Mt. Corrasi (code P) (Table 2). When the SMM in TPM was set to 70% and its variance to 30%, the latter population approached a threshold of significance under TPM (P = 0.042).

According to the modG‐W index, low values were recorded across all populations (Table 2), ranging from 0.137 (±SE 0.102) in *C. trinervis* (code T) to 0.171 (±SE 0.123) from *C. amazonum* of Mt. Corrasi (code P) (Table 2). These values indicate past reductions in population sizes for all groups, with values falling below the critical threshold of 0.68 (Garza & Williamson 2001).

### Phylogeographic survey

#### Plastid sequences

**Fig. 6:**
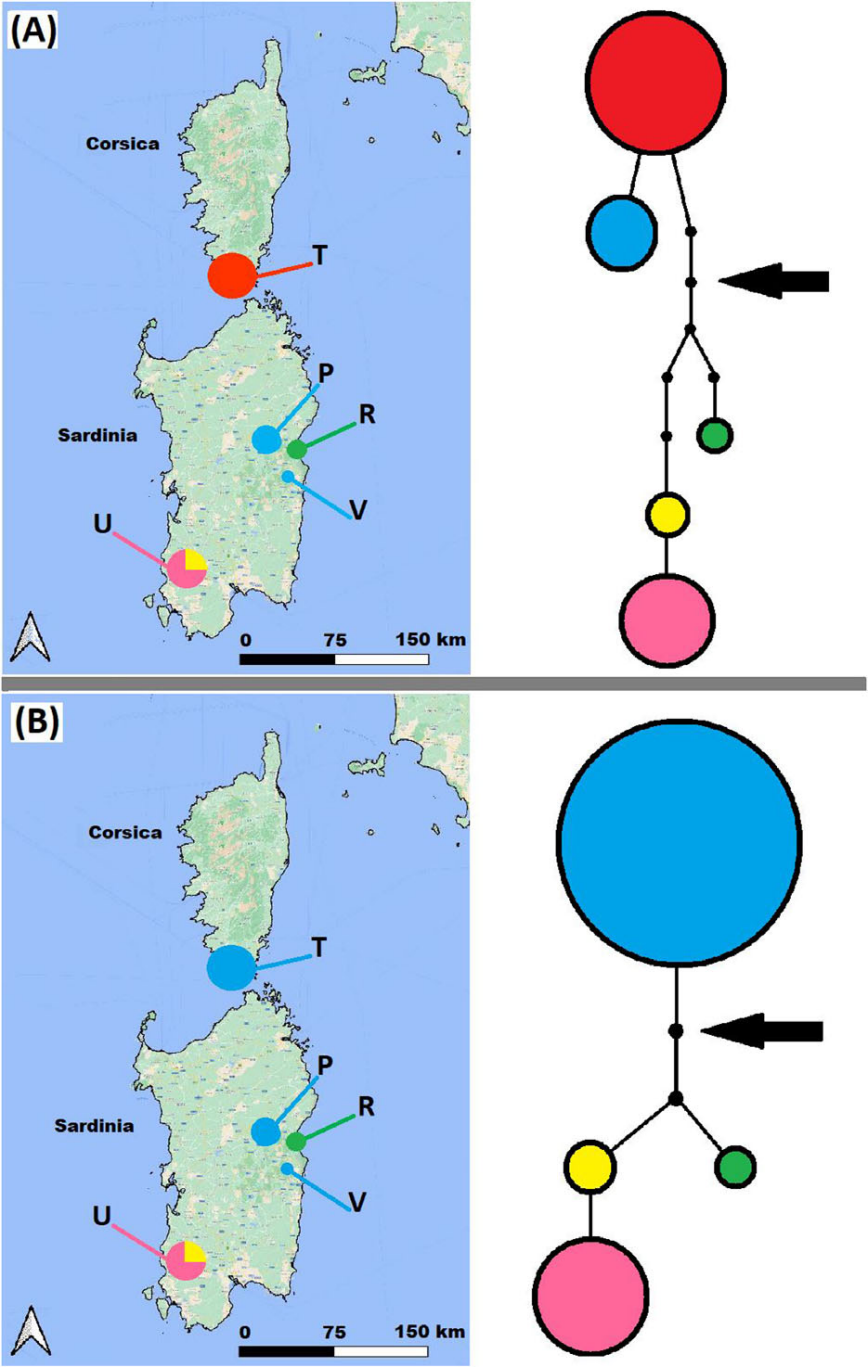
Geographical map displaying the distribution and frequency of haplotypes, alongside genealogical relationships derived through TCS analysis in *Centranthus* population in study. TCS analysis considers insertions as a fifth state (A), whilst excluding them (B); the size of the shape is proportional to the frequency of each haplotype black circles represent missing haplotypes (not sampled or extinct) and each line in the parsimony network represents one mutational step. The black arrow indicates the insertion point of the outgroup. For population legends and haplotypes information, refer to Table 1 and Appendix S1–Sheet 10.

A geographic map to assess the distribution and frequency of haplotypes is presented in Fig. 6. Among the six plastid markers utilised, the *mat*K gene (code V2) and the *acc*D‐*psa*I IGS (code P21) exhibited the highest variability (two SNPs; and one SNP, an indel, and a polyT sequence, respectively) (Appendix S1–Sheet 10). This was followed by the *rp*S15‐*ndh*F IGS (code P37), which had one SNP and an indel; the *trn*W^(CCA)^‐*trn*P^(UGG)^‐*psa*J IGS (code D19), with one SNP; and both the *trn*S^(GCU)^‐*trn*G^(UCC)^ IGS (code D20) and the *psb*A‐*trn*H^(GUG)^ IGS (code V4) each presenting one indel (Appendix S1–Sheet 10). Following the concatenation of the six alignment matrices, five haplotypes were detected when considering indels as a fifth state, or four, when scoring indels as missing data (Table 1, Fig. 6A,B, respectively). In this scenario, the haplotype from *C. trinervis* merges with the haplotype of *C. amazonum* from Mt. Corrasi and Mt. Perda. No haplotype is shared between populations, with the exception of a single case in *C. amazonum* (Gosollei versus Mt. Corrasi, codes U and P, respectively). Each geographic locality covered by our sampling exhibits at least one unique haplotype and, only the southern population of *C. pontecorvi* shows two haplotypes (Fig. 6, Table 1).

#### Genealogical relationships

The MP networks obtained do not contain ambiguities (loops) caused by possible homoplasy, which could lead to alternative genealogical pathways. The genealogical relationships are clearly depicted in Fig. 6. The MP network indicates close relationships between *C. trinervis* (red haplotype) and *C. amazonum* (blue haplotype) from Mt. Corrasi and Gosollei (codes P and V), distinguished by a unique indel of four nucleotides (Fig. 6A). When considering only SNPs (i.e. with the indel absent), the *C. trinervis* haplotype falls into the blue haplotype, which is characteristic of *C. amazonum* from Mt. Corrasi and Gosollei (codes P and V; Fig. 6B). From this group, through several intermediate haplotypes that are missing (not sampled or extinct), characteristic haplotypes for the two populations of *C. amazonum* from Codula di Luna (green haplotype) and *C. pontecorvi* from Mt. Perda (yellow and pink haplotypes) are identified. Overall, *C. amazonum*, as a specific taxon, demonstrates a greater variability of haplotypes, each being exclusive to its locality (Fig. 6). *Centranthus pontecorvi* exhibits the most genetically distant haplotypes and greater individual variability, since the population is represented by two haplotypes (yellow and pink), one of which is more frequent (pink, 75%). Utilising the outgroups to root the MP network reveals that the red haplotype of *C. trinervis* (considering the indel as a fifth state; Fig. 6A) or the blue haplotype of *C. trinervis*/*C. amazonum* from Mt. Corrasi and Gosollei (with the indel absent; Fig. 6B) appears to be the closest to the ancestral haplotype.

## DISCUSSION

### Nuclear microsatellites (nrDNA) versus plastid sequences (cpDNA)

In this study, the use of both plastid and nuclear DNA was crucial, as their inheritance mechanisms [maternal (seed) versus biparental (pollen)], and different mutation rates allowed the exploration of different evolutionary time scales in the analysis (Freeland *et al*. 2012; Gargiulo *et al*. 2019; De Castro *et al*. 2020a; Di Iorio *et al*. 2023). Specifically, plastid sequences, with their slower evolutionary rate and lack of recombination (Rousseau‐Gueutin *et al*. 2018), provide insights into historical seed dispersal and offer a perspective on the plant's evolutionary history, shedding light on phylogeographic relationships. This can assist in interpreting data obtained from variable nuclear populations markers, such as nuclear microsatellite markers (*e.g*. De Castro *et al*. 2016; De Castro *et al*. 2020b; Di Iorio *et al*. 2023). Indeed, nuclear microsatellites have been used to discern recent gene flow pattern, due to their high mutational rate (Marriage *et al*. 2009), providing an accurate view of genetic structure and population diversity, essential for evaluating potential conservation and protection measures (Gargiulo *et al*. 2019; Di Iorio *et al*. 2023).

### Population genetic survey

#### Genetic erosion and population bottlenecks

The results obtained from microsatellite analyses indicate that genetic erosion affects all examined populations of *Centranthus*, particularly *C. trinervis* and *C. amazonum* from Mt. Corrasi (codes T and P; Table 1). This is directly linked to the estimated effective population sizes (Ne) of these populations (Table 2). The data reveal a severe loss of genetic diversity in recent generations, which appears irreversible given the species' critical endemism (i.e. presence in only one or few populations) and the geographic barriers to dispersal. Genetic erosion is a well‐documented consequence of isolation, where gene flow remains restricted within a population (Frankham & Briscoe 2010; Freeland *et al*. 2012; Frankham *et al*. 2017; Freeland 2020), as observed in various taxa (e.g. *Asperula crassifolia* L., in Gargiulo *et al*. 2019; and/or Italian populations of *Cypripedium calceolus* L., Gargiulo *et al.* 2021). Demographic analyses suggest that the *C. trinervis* population has undergone bottleneck events both in recent and more distant time (Table 2). Additionally, older population reductions or bottlenecks were detected in Sardinian populations, including *C. amazonum* from Mt. Corrasi and *C. pontecorvi* (codes P and U; Table 2).

#### Conservation and genetic impact in *C. trinervis*

As previously suggested in the literature (Fridlender & Raynal‐Roques 1998; Montmollin and Strahm 2005; Bacchetta *et al*. 2008; Revaka *et al*. 2012), the *C. trinervis* population appears to be the most at risk among those studied (excluding the two Sardinian populations with sampling deficit; Table 1). This is related to: (1) its lowest genetic diversity or allelic richness (Table 1); (2) being the only known population of the species; and (3) its genetic distinction from other Sardinia populations (Figs. 3, 4). Bayesian inference analyses (Fig. 4) confirm that *C. trinervis* possesses a distinct genetic pool compared to other *Centranthus* populations. Additionally, PCoA (Fig. 3) reveals that *C. trinervis* individuals form a well‐defined cluster, genetically distant from other taxa. Therefore, given points (2) and (3), introducing individuals from other genetically similar populations to *C. trinervis* is entirely unfeasible. Should new populations be discovered, they must undergo thorough deep pre‐emptive population genetics analyses (Frankham & Briscoe 2010; Gargiulo *et al*. 2021).

Currently, the genetic diversity of *C. trinervis* remains a concern, despite reintroductions efforts from the same population (Table 1) (Revaka *et al*. 2012). These efforts have not mitigated the decline in genetic diversity and have, paradoxically, exacerbated it, potentially leading to inbreeding depression. Analysis indicates that introducing 11 individuals has reduced genetic diversity and increased the inbreeding coefficient, deviating from Hardy–Weinberg equilibrium expectations (Table 1). This is because these individuals are genetically similar, forming a distinct sub‐cluster in the PCoA analysis (Fig. 3). Bayesian clustering further confirms that introduced individuals have a unique genetic pool compared to native individuals (Fig. 5A). These observations necessitate a re‐evaluation of reintroduction strategies for critically endangered species, particularly when using genetically similar individuals. The evidence clearly shows that introducing closely related individuals can exacerbate the reduction in genetic diversity within already small natural populations (Freeland *et al*. 2012; Freeland 2020). As a result, the genetic diversity of *C. trinervis* is severely compromised, suggesting that this taxon may be considered genetically extinct, unless new populations are discovered to bolster the diminished genetic diversity without causing outbreeding depression (Freeland *et al*. 2012; Jamieson & Allendorf 2012; Frankham *et al*. 2014; Freeland 2020). Indeed, given that genetic erosion is now difficult, if not impossible, to reverse, factors that could accelerate the extinction of this species include: (1) climate change, with global warming advancing rapidly and causing significant, potentially destructive impacts on habitat maintenance; (2) the potential collection by tourists and botanophiles; and (3) indiscriminate land use for recreational and sporting activities, which have already seriously degraded the conservation status of *C. trinervis* on Mt. Trinité (Revaka *et al*. 2012; Pasta *et al*. 2017; Bacchetta *et al*. 2024).

#### Genetic structure and conservation of *C. amazonum* populations

The analysis of the remaining populations confirms that the lone *C. amazonum* individual from Gosollei (code V) clearly belongs to the same Bayesian cluster as the Mount Corrasi population (code P), although further insights due to the absence of additional specimens from Gosollei (Figs. 4 and 5). Similarly, despite limited sampling, the Codula di Luna population (code R) is part of the *C. amazonum* genetic pool but shows greater genetic distance from Mt. Corrasi, as indicated by both PCoA and Bayesian analyses (Figs. 3, 4, 5), highlighting its distinct genetic separation from other *C. amazonum* populations. Additionally, this population exhibits an unusual negative inbreeding coefficient (code R; Table 1), attributed to a high number of heterozygotes, which is atypical for small populations. This suggest that heterozygous individuals may prevail even in small populations; however, this should not be interpreted as a sign of population health (Gargiulo *et al*. 2021). This peculiarity may simply stem from the fact that the few remaining individuals are older remnants of a previously larger population, as also hypothesized by Bacchetta *et al*. (2008). However, it is evident that only the Mt. Corrasi population displays moderate genetic variability (code P; Table 1); but, even in this case, genetic erosion has led to a concerning situation, warranting prioritised conservation efforts. Given the species' limited population size, there is a tangible risk of extinction, although Fenu *et al*. (2024) confirm that there has been no continuous decline in population size over the years monitored. Despite the genetic affinities observed among some populations, no significant gene flow is evident (Appendix S1–Sheet 7), likely related to geographic distances and/or the absence of populations that could create genetic connectivity.

#### Conservation status and genetic health of *C. pontecorvi*

The newly discovered species in southwestern Sardinia, *C. pontecorvi* (code U), possesses a distinct genetic pool, separate from other *C. amazonum* and *C. trinervis* populations (Figs. 3 and 4). In term of conservation, this population appears to be in good health, maintaining high genetic diversity, despite historical bottleneck events (Tables 1 and 2). Given its unique genetic characteristics and isolation, a conservation strategy is essential, particularly in light of climate variations and potential human exploitation for scientific research and collection purposes. Notably, recent extreme climate events, with a strong trend towards tropicalisation (prolonged droughts and brief but intense rainfall), may impact habitats supporting these populations. This could lead to collapses and erosion of rock faces following torrential rains. Additionally, due to increasingly extended dry seasons, there has been an observed rise in grazing by mouflons and goats on individuals in more accessible sites, a phenomenon previously reported by Bacchetta *et al*. (2008).

### Phylogeographic survey

#### Haplotype fixation and genealogical relationships in *C. amazonum* and *C. trinervis* populations

The data obtained from analysing mutations across six plastid markers (Appendix S1–Sheet 10) reveal the presence of various largely fixed haplotypes, regardless of whether insertions are considered informative (Table 1, Fig. 6). The presence of haplotypic fixation can be attributed to the intrinsic nature of their evolutionary state. Indeed, the population genetics (nrSSRs) implemented here indicates that genetic drift and bottleneck events have substantially reduced the original population diversity and, consequently, the haplotypic variability. In this context, the genealogical relationships inferred from haplotypes in the studied *Centranthus* populations confirm the genetic identity of the *C. amazonum* specimen from Gosellei with the population from Mt. Corrasi (codes V and P; Table 1), both when considering insertions as informative or missing state (Fig. 6). Population genetic data further support this result (Figs. 3 and 4). The haplotype B (blue; Fig. 6), characteristic of *C. amazonum* for both Mt. Corrasi and Gosollei (codes V and P; Table 1), is also shared with the *C. trinervis* population when considering the insertions as assent data (see Fig. 6A). In genealogical reconstruction using a parsimonious approach, insertions require careful evaluation due to potential homology concerns (Provan *et al*. 2001; De Castro *et al*. 2022). Here, in examining the parsimonious network with insertions weighted evolutionarily, it appears that *C. trinervis* possesses a unique diagnostic haplotype (red) related to that of the two *C. amazonum* locations (blue; codes P and U; Fig. 6A). Given the lack of intermediate haplotypes and their differentiation by a single insertion, this supports an extremely close genetic relationship between these two taxa. If the insertion is considered informative, it suggests that the haplotype in the *C. trinervis* population gave rise to the diagnostic haplotype for the two *C. amazonum* populations (codes P and V; Fig. 6A). Therefore, in the genealogical relationships, even considering insertions, a close evolutionary proximity of these two haplotypes and the taxa associated with them is confirmed (Fig. 6A). Finally, from a common ancestor through intermediate haplotypes (extinct or unsampled), the haplotypes of Codula di Luna and M. Perda would have been generated through various evolutionary steps (codes R and U; Fig. 6). This observation is consistent across both genealogical reconstructions, regardless of insertions being considered informative (Fig. 6A, B).

#### Haplotype variability and genetic isolation in *C. pontecorvi*

According to *C. pontecorvi*, this population exhibits greater haplotypic variability compared to other populations. It comprises two haplotypes that are consistently diagnostic for the population under study (Fig. 6, Table 1). Examination of genealogical relationships indicates that this population is the most divergent among those studied and shares a closer relationship with the *C. amazonum* population from Codula di Luna (code R, Table 1; Fig. 6) through hypothetical intermediate haplotypes that might have been lost. Factors potentially contributing to the patterns observed in this and other populations include: (1) small population sizes, where genetic drift has homogenised the most prevalent haplotypes; (2) possible bottlenecks; (3) founder effect; or (4) gaps in sampling. The distinctiveness of this population is further supported by population genetic data, showing that it is genetically distant from the *C. amazonum* populations and harbours a unique nuclear genetic pool (Figs. 3 and 4).

#### Range distribution and loss of genetic diversity

The data obtained, when cross‐referenced with nuclear results (i.e. population genetics), suggest that in Sardinia and Corsica, the species under study historically exhibited greater genetic variability, which has since been lost through range fragmentation and population depletion. It is crucial to note that, given the relict nature of the populations analysed, the haplotypes currently fixed may once have occurred with higher frequency within these populations or across a broader range that no longer exists. Therefore, it is reasonable to consider that genealogical inferences in this specific case may be biased by the fact that we observe only residual haplotypes in remnant populations, derived from a genetic variability and distribution range that were likely more extensive in the past.

## CONCLUSION AND IMPLICATIONS FOR CONSERVATION

The species investigated are relic taxa that now occupy only a small fraction of their original range, as evidenced by both genetic data obtained and supported by geological, climatic, and environmental factors that have led to a restriction of the range (Bacchetta *et al*. 2024). While the environment has contributed to the genetic decline of these populations, their inaccessibility has allowed the survival of the rare populations of *Centranthus* from sect. *Nervosae*, limiting both human impact, such as cultivation or grazing, and the progression of plant succession towards different climax communities (*e.g*. forests) (Fenu *et al*. 2024). Two key concepts can explain the reduced genetic variability and lack of gene flow in the taxa analysed. First, these taxa are specialised for local ecological conditions, and natural selection has reduced and will continue to homogenise genetic variation, as maladapted alleles are promptly eliminated (Freeland *et al*. 2012; Freeland 2020). Second, the small population size, coupled with bottlenecks or founder effects, have led to a rapid increase in inbreeding, with all its negative impacts on fitness, genetic drift and genetic variability. Consequently, genetic variability has rapidly decreased or remained low within these populations, which will continue to diverge from each other until they form distinct genetic units, increasingly vulnerable to extinction.

## Author contributions

ODC: Conceptualization, Methodology, Molecular laboratory work, Software Formal analysis, Data curation, Writing – Original Draft, Review and Editing, Funding acquisition. EDI: Molecular laboratory work. CP: Sampling, Review and Editing. GB: Sampling, Review and Editing, Funding acquisition. BM: Data curation, Review and Editing. All authors contributed to previous versions of the manuscript and approved the final version for submission.

## Funding information

This study was supported by l'Office de l'Environnement de la Corse (OEC) and le Conservatoire Botanique National de Corse (CBNC) (Convention n°20/05, Département: CBNC, Service: 33; 20/11/2020).

## Conflict of interest

The authors declare that they have no known competing financial interests or personal relationships that could appear to influence the work reported in this paper.

## Supporting information


**File S1.** Alignments of *trn*W^(CCA)^‐*trn*P^(UGG)^‐*psa*J IGS (D19) haplotypic sequences (fasta format). Order of the samples as reported in Appendix–sheet 10.

**File S2.** Alignments of *trn*S^(GCU)^‐*trn*G^(UCC)^ IGS (D20) haplotypic sequences (fasta format). Order of the samples as reported in Appendix–sheet 10.

**File S3.** Alignments of *acc*D‐*psa*I IGS (D21) haplotypic sequences (fasta format). Order of the samples as reported in Appendix–sheet 10.

**File S4.** Alignments of *rpS*15‐*ndh*F IGS (P37) haplotypic sequences (fasta format). Order of the samples as reported in Appendix–sheet 10.

**File S5.** Alignments of *mat*K(−3′) gene (V2) haplotypic sequences (fasta format). Order of the samples as reported in Appendix–sheet 10.

**File S6.** Alignments of *psb*A‐*trn*H^(GUG)^ IGS (V4) haplotypic sequences (fasta format). Order of the samples as reported in Appendix–sheet 10.

**Appendix S1.** Sheet 1: Information on loci used in the populations of *Centranthus* sect. *Nervosae*. Sheet 2: Matrix of SSR loci used in populations of *Centranthus* sect. *Nervosae*. Sheet 3: Information on the 66 plastid molecular markers screened in populations of *Centranthus* sect. *Nervosae*. Two individuals per population were analysed, except for the V locality, which was represented by a single individual (Table 1). Sheet 4: Information on the six plastid markers used in populations of *Centranthus* sect. *Nervosae*. Sheet 5: Null allele frequencies (NAF) were calculated using both the FreeNA software (Chapuis & Estoup 2007) with 10,000 bootstrap replications and the ML‐NULLFREQ software (Kalinowski & Taper 2006). Sheet 6: Probability tests for Hardy–Weinberg proportions and F_IS_ values per locus/population of *Centranthus* sect. *Nervosae*, as implemented in GenePop. Sheet 7: Pairwise genetic distance matrix of F_ST_ with P‐values, and matrix of Slatkin linearized F_ST_ [F_ST_/(1—F_ST_)] for populations of *Centranthus* sect. *Nervosae*, as implemented in Arlequin. Sheet 8: Pairwise genetic distance matrix of Da (Nei et al. 1983) for individuals of *Centranthus* sect. *Nervosae*, as implemented in Populations software. Sheet 9: The most adequate value for the number of clusters (K) in populations of *Centranthus* sect. *Nervosae*, obtained using the method of Evanno et al. (2005), as implemented in STRUCTURE HARVESTER. Sheet 10: Haplotypes detected in the six plastid markers across the five populations of *Centranthus* sect. *Nervosae*. Refer to Fig. 6 for their geographic distribution on the map. Sheet 11: GenBank accession codes for the plastid molecular markers obtained for the two outgroup species (*Centranthus calcitrapae* and *C. macrosiphon*) used in analysing genealogical relationships among the five populations of *Centranthus* sect. *Nervosae*.

## Data Availability

The datasets generated during and/or analysed during the current study are available from the corresponding author on reasonable request.
